# Data on the effects of low iron diet on serum lipid profile in HCV transgenic mouse model

**DOI:** 10.1016/j.dib.2017.03.020

**Published:** 2017-03-18

**Authors:** Alice Conigliaro, Viviana Costa, Rosario Amato, Carmine Mancone

**Affiliations:** a Dipartimento di Biotecnologie Cellulari ed Ematologia, Sapienza University of Rome, Rome 00185, Italy; b Rizzoli Orthopedic Institute, Palermo, Italy, Innovative Technological Platforms for Tissue Engineering, Theranostic and Oncology, Palermo, Italy; c Department of “Scienze della Salute”, University”Magna Græcia” of Catanzaro, Viale Europa, Catanzaro, Italy

**Keywords:** Iron depletion, Triglycerides, Low density lipoproteins

## Abstract

Here, we presented new original data on the effects of iron depletion on the circulating lipid profile in B6HCV mice, a murine model of HCV-related dyslipidemia. Male adult B6HCV mice were subjected to non-invasive iron depletion by low iron diet. Serum iron concentration was assessed for evaluating the effects of the dietary iron depletion. Concentrations of circulating triglycerides, total cholesterol, Low Density Lipoproteins (LDLs), High Density Lipoproteins (HDLs) were analyzed and reported by using stacked line charts. The present data indicated that low serum iron concentration is associated to i) lower serum triglycerides concentrations and ii) increased circulating LDLs. The presented original data have not been published elsewhere.

**Specifications Table**

**Table:** 

Subject area	*Biology*
More specific subject area	*Biochemistry, Genetics and Molecular Biology*
Type of data	*Table, figures, Text file.*
How data was acquired	*Analyses were performed on an automated haematology analyser using mouse-specific algorithms and parameters (ProCyte DX IDEXX and Abaxis VetScan5).*
Data format	*Raw, Analyzed*
Experimental factors	*B6HCV transgenic mice were from Markus Heim laboratory at Department of Biomedicine University Hospital of Basel*
Experimental features	*Iron and lipids concentrations were measured in serum samples of B6HCV mice fed low iron diet for thirty days.*
Data source location	*Palermo, Italy*
Data accessibility	*Data are available with this article*

**Value of the data**

• The presented data indicated that low-iron diet i) reduced serum triglycerides concentrations and ii) increased levels of circulating LDLs in HCV transgenic mouse model. No differences have been found on cholesterol and HDLs concentrations in low iron feed mice respect to controls.
• These data are the further evidence on the interplay between the iron and lipid metabolism.
• The data in this article could be useful to optimize further clinical trials to evaluate the potential therapeutic effect of dietary iron depletion on dyslipidaemia in patients with Hepatitis C infection and hepatic iron overload.

## 1 Data

**Fig. 1:**
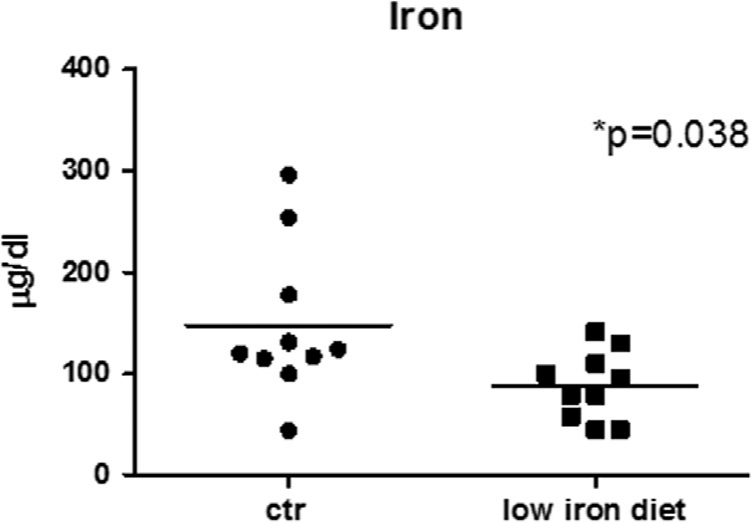
Serum iron concentration in B6HCV mice fed the normal (*n*.10) and low (*n*.10) iron diet. *p* value is indicated; * significant difference. For details see Methods section.

**Fig. 2:**
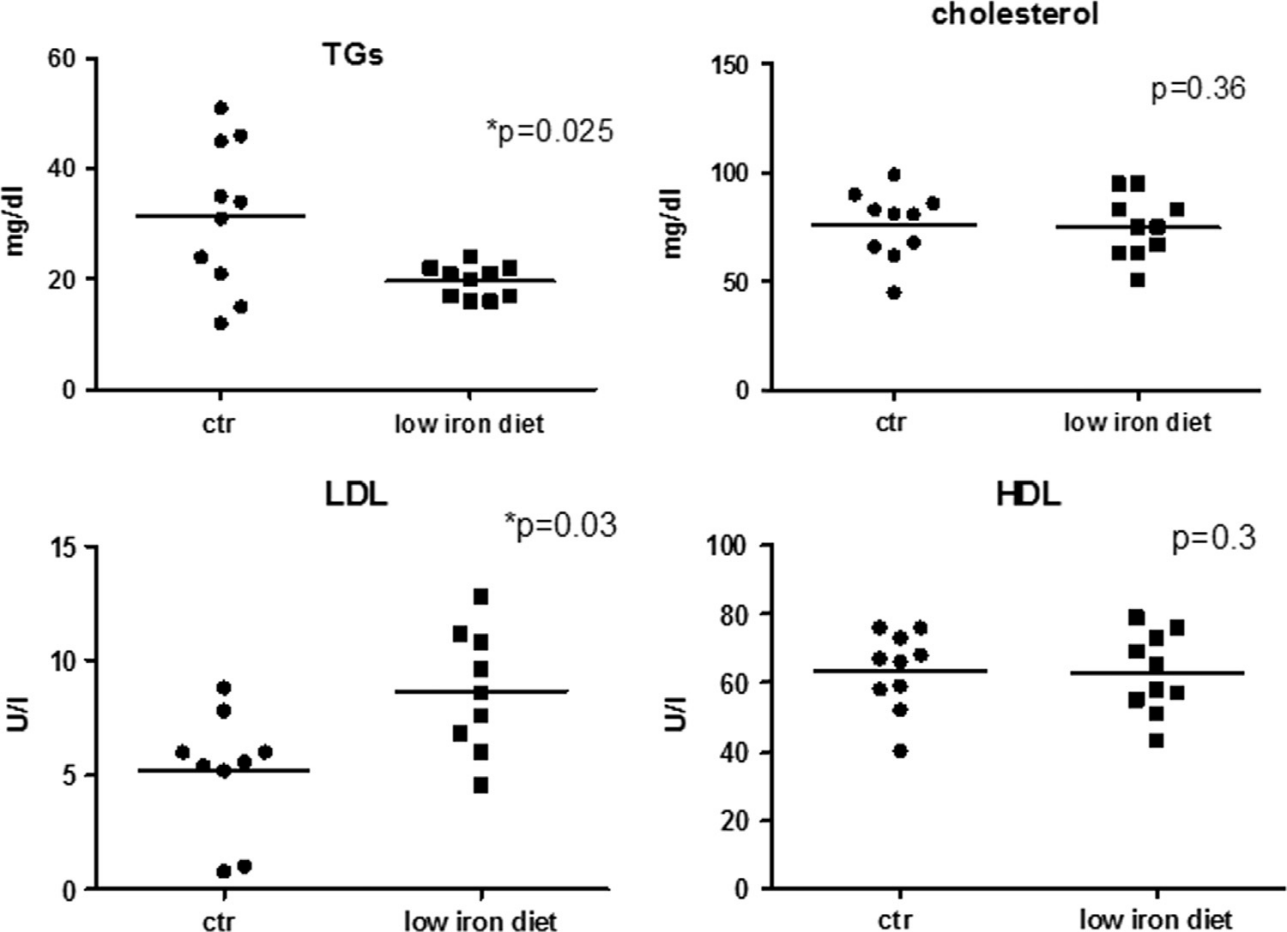
Quantitative analysis of the indicated classes of lipids in the serum samples of B6HCV mice fed normal (*n*.10) and low (*n*.10) iron diet. *p* values are indicated; * significant differences. For details see Methods section.

Mice were fed a low-iron diet for thirty days and reduction in blood iron concentration was assessed by biochemical analysis (Fig. 1). During the treatment B6HCV mice did not show any sign of distress. Compared with control group, low iron diet resulted in a significant reduction in circulating triglycerides (TGs), while there was no change in total cholesterol concentration (Fig. 2). Moreover, mice fed the low-iron diet showed increased levels of circulating LDLs respect to control group; no differences were observed in the concentrations of HDLs (Fig. 2). All the obtained results are summarized in Table 1.

## 2 Experimental design

To date, many evidences showed that iron overload plays a causal role in the hepatic fat accumulation. In particular, studies on HCV (hepatitis-C-virus) transgenic mice fed an excess-iron diet demonstrated that hepatic iron overload exacerbates the imbalance of lipid metabolism by both lipogenesis activation and reduced oxidation activity of fatty acids [1], [2]. Moreover, HCV-induced iron accumulation contributes to liver steatosis by the inhibition of β-lipoproteins mediated fat export, thus leading hypobetalipoproteinemia [3], [4]. In this study, male B6HCV-transgenic mice [5] expressing the HCV polyprotein were fed a low-iron diet or a control diet for thirty days. Mice in each group were assessed for blood iron concentration and circulating lipid profiling (triglycerides, total cholesterol, Low Density Lipoproteins (LDLs), High Density Lipoproteins (HDLs)).

## 3 Materials and methods

B6HCV transgenic mice were from Markus Heim laboratory at Department of Biomedicine University Hospital of Basel [5]. Mice were bred and maintained in accordance with the institutional guidelines of the University of Palermo Animal Care Committee. The experimental procedures have been communicated to the Ministry of Health in accordance with the ministerial directive at the time of the experiments: legislative decree 116/92, annex 4 to the circular of the Ministry of Health n. 8 of 1994. No suffering was inflicted on the animals before euthanasia. Twenty male B6HCV (6 months old) equally divided into 2 groups, were fed a low-iron diet (ssniff EF R/M iron deficient diet) or relative control diet. After one month, mice were killed by CO_2_ asphyxiation and blood collected by post-mortem cardiac puncture. No animals became severely ill or died prior to the experimental endpoint. Euthanasia in mice was performed by carbon dioxide asphyxiation.

Blood biochemical analysis were performed on an automated hematology analyzer using mouse-specific algorithms and parameters (ProCyte DX IDEXX and Abaxis VetScan5). Total cholesterol was measured by the CHOD-PAP method adapted to automated analyzer (Chem 400), non-complexed HDL was measured using the CHOD-PAP method. For triglyceride determinations GPO-PAP method was used. Quantitative values are expressed as mean±SD (Table 1). Data between two groups (control and low iron diet) were compared by Student׳s *t*-test. The statistical significance of correlation was determined by the use of a simple regression analysis. A *P* value of 0.05 was considered to be significant.

## Funding

This work was supported by grants from MIUR Ministero dell’Università e Ricerca Scientifica (FIRB 2012, codice unico progetto B81J12003390001: RBFR12NSCF-001, RBFR12NSCF-002, RBFR12NSCF-003).

## Figures and Tables

**Table 1:** Biochemical results of B6HCV mice fed the normal (Ctr) and low iron diet (Lid).

	**Iron mg/dl**		**Triglycerides mg/dl**		**Total cholesterol mg/dl**		**LDL U/l**		**HDL U/l**	
	**Ctr**	**Lid**	**Ctr**	**Lid**	**Ctr**	**Lid**	**Ctr**	**Lid**	**Ctr**	**Lid**
**Mean**	117,8	77,60	31,67	19,17	78,29	75,00	5,057	8,480	65,29	63,33
**Minimum**	100,0	45,00	12,00	16,00	45,00	51,00	0,8000	6,000	40,00	43,00
**Maximum**	131,0	110,0	46,00	24,00	99,00	95,00	8,800	11,20	76,00	79,00
**Std. Deviation**	10,42	26,63	13,38	3,371	17,79	15,80	3,093	2,373	12,84	13,71
